# Measuring potential effects of the developmental burden associated with the vertebrate notochord

**DOI:** 10.1002/jez.b.23032

**Published:** 2021-03-10

**Authors:** Satoko Fujimoto, Kaori Yamanaka, Chiharu Tanegashima, Osamu Nishimura, Shigehiro Kuraku, Shigeru Kuratani, Naoki Irie

**Affiliations:** ^1^ RIKEN, Center for Biosystems Dynamics Research Kobe Japan; ^2^ Department of Biological Sciences The University of Tokyo Tokyo Japan; ^3^ Universal Biology Institute The University of Tokyo Tokyo Japan

**Keywords:** developmental burden, evolution, gene expression profile, notochord, phylotypic period

## Abstract

The notochord functions primarily as a supporting tissue to maintain the anteroposterior axis of primitive chordates, a function that is replaced entirely by the vertebral column in many vertebrates. The notochord still appears during vertebrate embryogenesis and plays a crucial role in the developmental pattern formation of surrounding structures, such as the somites and neural tube, providing the basis for the vertebrate body plan. The indispensable role of the notochord has often been referred to as the developmental burden and used to explain the evolutionary conservation of notochord; however, the existence of this burden has not been successfully exemplified so far. Since the adaptive value of target tissues appears to result in the evolutionary conservation of upstream structures through the developmental burden, we performed comparative gene expression profiling of the notochord, somites, and neural tube during the mid‐embryonic stages in turtles and chicken to measure their evolutionary conservation. When compared with the somites and neural tube, overall gene expression profiles in the notochord showed significantly lower or merely comparable levels of conservation. However, genes involved in inductive signalings, such as the sonic hedgehog (*Shh*) cascade and the formation of functional primary cilia, showed relatively higher levels of conservation in all the three structures analyzed. Collectively, these results suggest that *shh* signals are critical as the inductive source and receiving structures, possibly constituting the inter‐dependencies of developmental burden.

## Research Highlights

Potential evolutionary effects toward notochord by developmental burden was evaluated by Laser Micro Dissection RNAseq (LMDseq). Notochord was less conserved than neural tube and somites; however, genes in sonic hedgehog (shh) signaling cascade was found to be evolutionarily conserved (not only in notochord but also in somites and neural tube). These results suggest that Shh signals are critical as the inductive source and receiving structures, possibly constituting the inter‐dependencies of developmental burden. Further studies that directly measure the burden required to verify the hypothesis are awaited.

## Graphical abstract

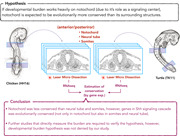

## 1 INTRODUCTION

The vertebrate notochord is regarded as one of the best examples to justify Haeckel's recapitulation theory (Haeckel, 1866), or von Baer's law (von Baer 1828), as this temporal embryonic structure seemingly reflects the ancestry of chordates and disappears afterward when the embryo develops into an adult, which appear as “higher” vertebrates. Debates for over century‐long (Richardson & Keuck, 2002), together with comparative transcriptomic studies (Domazet‐Loso & Tautz, 2010; Duboule, 1994; Hazkani‐Covo et al., 2005; H. Hu et al., 2017; Irie & Kuratani, 2011; Irie & Sehara‐Fujisawa, 2007; Kalinka et al., 2010; Z. Wang, Pascual‐Anaya, et al., 2013; Xu et al., 2016) clarified that the recapitulation theory and the early conservation model (reviewed in Kalinka & Tomancak, 2012) could not be accepted as a whole to explain the evolutionary tendencies of embryos. In brief, these studies indicated that the mid‐embryonic organogenesis period was the most evolutionarily conserved stage during development, rather than the earliest developmental stage. However, it has to be noted that an early conservation tendency can still be observed for some morphological traits (Abzhanov, 2013; Nagashima et al., 2009) and chromatin accessibilities (Uesaka et al., 2019), especially during the stages after the highly conserved mid‐embryonic period. More importantly, these studies do not necessarily refute mechanisms that support the recapitulation theory and the early conservation model (Garstang, 1922; Riedl, 1978; Wimsatt, 2007; reviewed in Irie, 2017). Developmental burden (Riedl, 1978), for example, predicts that late embryogenesis likely depends on earlier developmental events, which, in turn, lead to the evolutionary conservation of earlier processes (Irie & Kuratani, 2014).

No studies have been performed so far to directly measure the strength of developmental burden (Alan, 2014), and it is still way beyond the scope of this study; however, an indirect way of testing of the concept would be to quantify and evaluate the evolutionary conservation of embryonic structures that appear to be accompanied by a strong developmental burden. The notochord, for example, would make a good example to test this idea, as it appears in most of the chordate embryos whether or not it persists as an adult organ (Kuratani, 2017). The notochord is known to play a crucial role in developing its surrounding structures, such as the neural tube and somites (Kuratani & Ota, 2008), and this signaling dependency could lead to the evolutionary conservation of the notochord through developmental burden. In addition, the notochord appears to be more important for normal vertebrate embryogenesis than in protochordates (cephalochordates and urochordates), as no vertebrates skip the development of notochord, while some of the tailless ascidian species abbreviate its development (Jeffery & Swalla, 1992). Notably, not only the notochord but also the tail muscles and pigmented sensory organs are also lost in these tailless ascidians (Jeffery & Swalla, 1992). This suggests that the notochord is more heavily burdened in vertebrates than in protochordate (Kuratani, 2017), so vertebrates would be suitable for testing the possible effect of developmental burden. In this study, we searched for a possible sign of developmental burden by analyzing conserved molecular components of the notochord, somites, and neural tube between turtles and chicken. These species were selected since they show marked resemblance in anatomical features during the conserved mid‐embryonic phase, despite their remarkably different adult phenotypes and lineages that led to their species splitting around 250 million years ago (  Z. Wang, Pascual‐Anaya, et al., 2013). In addition to the availability of genomes and embryos, the evolutionarily distant relationship between these species was expected to provide a higher resolution for distinguishing conserved/diverged genetic profiles. For evaluating the conservation of specific structures, we measured the similarity of the gene expression profiles of the target structures between the different species, as in previous studies (H. Hu et al., 2017; Irie & Kuratani, 2011; Kalinka & Tomancak, 2012). Given the existing developmental burden, which plays a major role in the conservation of developmental burden around the notochord, it is expected that the genes involved in signaling dependencies of the developmental burden to be conserved.

## 2 MATERIALS AND METHODS

### 2.1 Animal care and use

Animal care and experimental procedures were conducted in strict accordance with the guidelines approved by the Animal Experiments Committee of RIKEN (H16‐10) and the University of Tokyo (approval ID: 14‐03, 16‐2). All efforts were made to minimize suffering. Individual animals and embryos were selected blindly from wild types.

### 2.2 Embryo sampling and imaging

**Figure 1:**
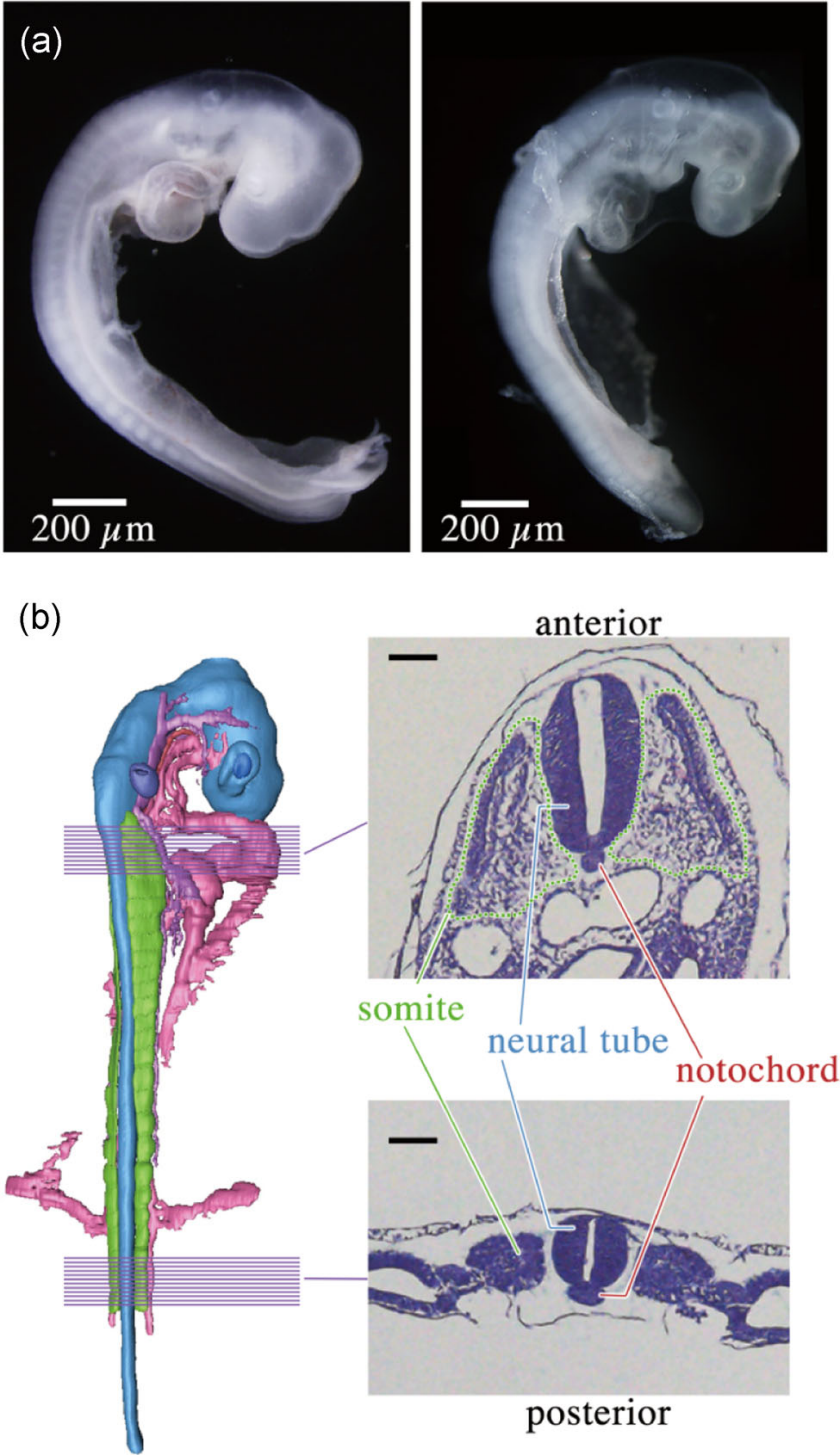
Embryos and structures targeted for the laser‐micro dissection‐based RNAseq. (a) Stage HH16 of the chicken embryo (left) and stage TK11 of turtle embryo (right). Images were modified and adapted from our previous study. (b) 3D reconstructed image of the chicken embryo at stage HH16 made by Avizo. Only neural tissues (blue), somites (green), notochord (red), and blood vessels (pink) are shown. Purple lines represent target anteroposterior levels. Representative images (hematoxylin and eosin‐stained) of anterior and posterior sections are shown on the right. As anterior somites have already started to differentiate in this stage, somite‐derived regions were also collected (green‐dashed line). Scale bars = 100 μm [Color figure can be viewed at wileyonlinelibrary.com]

Fertilized chicken eggs and soft‐shell turtle eggs were purchased from local farms in Japan during the breeding season of Chinese soft‐shelled turtles (mid‐June to early July). Eggs were incubated and collected at stage HH16 for chicken and TK11 for turtles. Amniotic membranes were removed before messenger RNA (mRNA) extraction. Three biological replicate samples were created. Images of embryos (Figure 1a) were taken using optical microscopy (Leica).

### 2.3 Laser microdissection

After treating staged embryos with RNAlater (Thermo Scientific Fisher) for 5 min, embryos were soaked into optimal cutting temperature compound (O.C.T. compound, Tissue‐Tek). Surrounding positions of target somites (first to third somites from the anterior‐most, and first to third somites from the posterior‐most) were labeled with red (colored with food coloring) O.C.T. compound under stereoscopic microscopy and frozen on dry ice. Frozen samples were sectioned using a cryostat (with 12‐μm thick), and sections with red marks were placed on a membrane slide NF 1.0 PEN (ZEISS #415190‐9081‐000) for further analysis. After morphologic identification by optical microscopy (ZEISS), the notochord, somites (both left and right sides), and neural tube regions were microdissected with a PALM MicroBeam laser Ver.4.3 (ZIESS), and total RNA of these laser‐dissected sections was collected using Agencourt RNACleanXP (#A63987; BECKMAN). As anterior somites are more differentiated than posterior somites, regions that correspond to somite‐derived structures, such as the dermatome, myotome, sclerotome, and syndetome were also targeted. The pairs of anterior and posterior samples of each tissue were collected from the same embryo.

### 2.4 RNA sequencing

The quality of the extracted RNA samples was checked with Bioanalyzer (Agilent). Whole‐transcriptome amplification was performed using the QUARTZ‐seq method (Sasagawa et al., 2013). The amplified complementary DNA was then sheared to generate fragments of 150–200 bp in length using Covaris E220 under the following conditions: duty factor 10%, peak incident power 175 W, 100 cycles per burst, and treatment time 600 s. Sequencing libraries were constructed using the KAPA Library Prep Kit (KAPA Biosystems) and single‐index Illumina TruSeq compatible adaptors. The cycle number of polymerase chain reaction amplification was determined for individual libraries (Tanegashima et al., 2018). DNA sequencing was performed on HiSeq 1500 (Illumina) in high‐output mode, and single‐end reads of 100 nt were obtained. The quality of the deep RNA sequencing reads was assessed with FastQC (v.0.10.1), and the mapping statistics of each sample are listed in Table S1.

### 2.5 Gene expression analysis

Genomes (Gallus_gallus.GRCg6a.dna_rm.toplevel.fa, Pelodiscus_sinensis.PelSin_1.0. dna_rm.toplevel.fa), gene transfer format (GTF) files, and other annotation files of *Pelodiscus sinensis* (Z. Wang, Pascual‐Anaya, et al., 2013) and *Gallus gallus* were downloaded from the Ensembl database (ver. 98). Sequences of mitochondrial genomes in each species were removed before mapping RNAseq reads to each species‐specific genome. Mapping of RNAseq reads was performed using the Hisat2 (ver.2.0.5) program (Kim et al., 2019), and expression levels for each gene were estimated by referring to the GTF file of each species (ensembl ver. 98), using the StringTie (v.1.3.5) software (Kovaka et al., 2019). Evolutionary conservation of gene expression profiles was performed either with 1:1 orthologs between chicken and turtle (12,279 orthologs), or ortholog‐group‐based comparisons (11,302 ortholog groups). To avoid unwanted bias by excluding paralogs and lost genes in 1:1 ortholog‐based comparisons, comparisons based on ortholog groups were also utilized to confirm the conclusion analyses. In this ortholog group method, ortholog groups were identified by orthoMCL (Li et al., 2003) as previously described [see Supplementary file of H. Hu et al., (2017)]. Expression levels of detected genes are summarized in table format and provided as Supporting Information Data S1.

### 2.6 Extraction of genes involved in the shh signaling pathway

Chicken genes with gene ontology (GO) term GO:0007224 (smoothened signaling pathway), and turtle orthologous counterparts (defined by 1:1 orthologs), were defined as genes involved in the shh signaling pathway, or shh‐related genes. Fifty‐six genes were identified as shh‐related genes in the chicken genome. Gene expression data obtained by RNAseq for stage TK27 turtles and stage HH38 chicken were obtained from previously published data (Z. Wang, Pascual‐Anaya, et al., 2013).

### 2.7 Statistical tests

ɑ of .05 were regarded as statistically significant throughout the study, unless otherwise specified. GSEABase R package (ver. 1.36) was used to analyze the GO terms downloaded from the Ensembl database (ver. 98).

## 3 RESULTS

### 3.1 Notochord was not the most conserved structure at the transcriptomic level

To minimize the potential bias from differences in the extent of development and/or differentiation (e.g., heterochronic shifts), we focused on the most evolutionarily conserved developmental stages between turtles (TK 11 for *P. sinensis*) and chicken (HH 16 for *G. gallus*) that have been identified in previous studies (H. Hu et al., 2017; Irie & Kuratani, 2011;  Z. Wang, Pascual‐Anaya, et al., 2013) (Figure 1a). These stages are also considered a potential phylotypic period (Richardson et al., 1998) for vertebrates, as they retain basic anatomical features shared between vertebrates (Irie et al., 2018; H. Hu et al., 2017; Z. Wang, Pascual‐Anaya, et al., 2013). In addition to developmental stages, anteroposterior (AP) positions within the embryo were also adjusted to minimize unwanted bias, as anterior somites are more differentiated than those in posterior regions. For this purpose, two AP levels (anterior‐most 1–3 somite level and posterior‐most 1–3 somite level) were targeted for our study. Gene expression profiles of the notochord, neural tube, and somites in these levels were detected by laser microdissection (LMD) followed by RNAseq **(**Figure 1b, see also Section 2 and Table S1).

**Figure 2:**
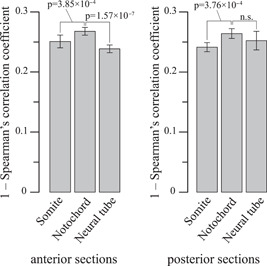
Evolutionary conservation of gene expression profiles in the notochord, neural tube, and somites between chicken and turtles. Evolutionary distances between gene expression profiles of chicken and turtles were evaluated by 1 – Spearman correlation coefficients of 1:1 orthologs. Bar plots on the left represent sections from the anterior level, and bar plots on the right represent sections from the posterior level. *N* = 3. Different structures were dissected from the same individual for each biological replicate. Error bar: *SD*, *p* values: Dunnett's test (two‐tailed)

Based on our comparative analysis of all 1:1 orthologous genes expressed in the target structures, we found that both the anterior and posterior notochord have lower or comparable levels of conservation when compared with neural tube and somite conservation (Figure 2, Supporting Information Data S1). The same tendency was also observed for results obtained by ortholog group‐based methods (H. Hu et al., 2017) that incorporate expression levels of genes potentially lost and duplicated (Figure S1, see also Section 2). As similarity in overall gene expression profiles is considered to reflect the similar composition of homologous cells, our results imply that developmental burden, if it exists, is not strong enough to keep cells in the notochord more conserved than those in neural tube and somites.

### 3.2 Conserved expression of shh‐related genes

**Figure 3:**
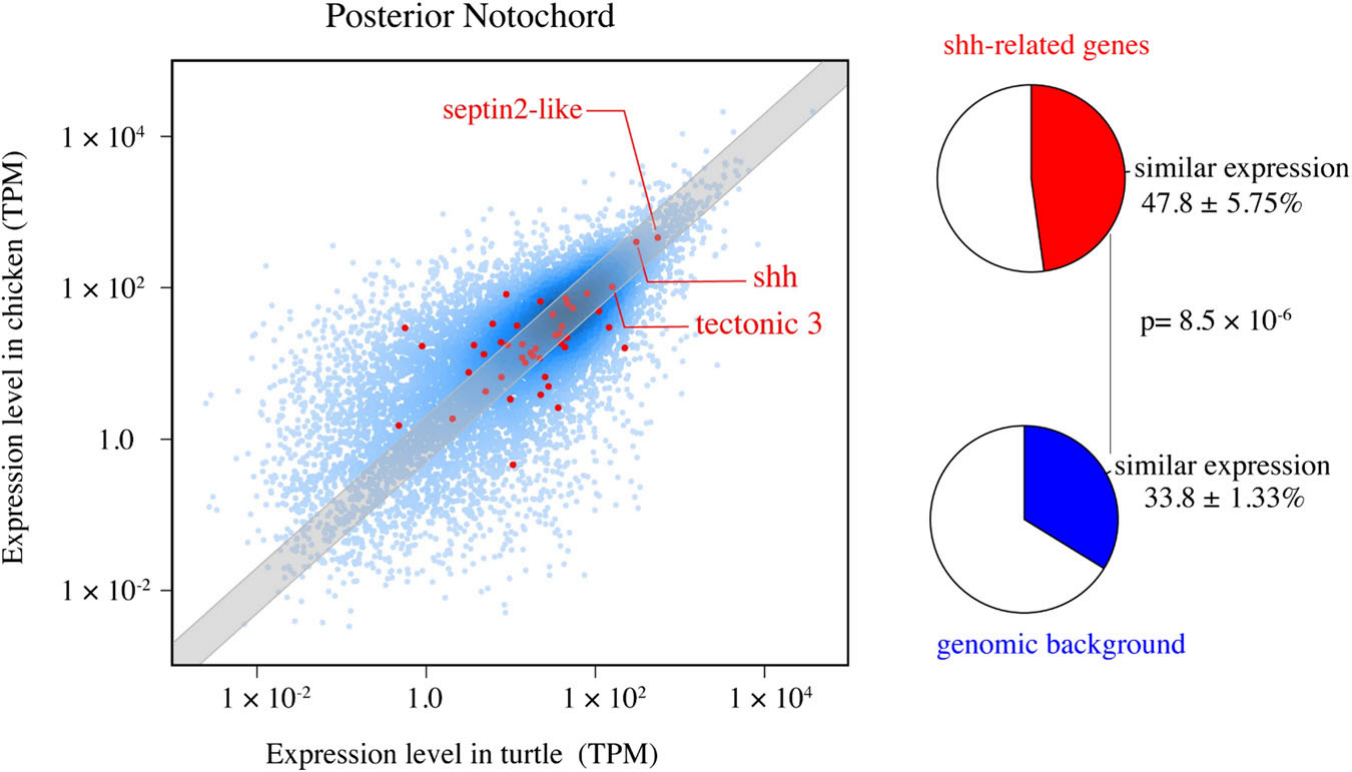
Expression levels of shh‐related genes in the posterior notochord. Left: Gene expression levels (TPM) in posterior notochords of chicken and turtles are shown as a scatter plot. Shh‐related genes (46 genes) are colored in red, and other genes in the genomic background are colored in light blue (12,233 genes). Each plot represents average expression levels of biological replicates within each species. The gray zone represents signal ratio chicken–turtle less than twofold. Right: Pie charts represent the ratio of shh‐related genes within the twofold range (up), and the ratio of genomic background (down). Deviations in ratios represent *SD*. The differences in the ratio of genes within the twofold range and genes outside the twofold range were statistically significant between shh‐related genes and the genomic background (Student's *t* test, *n* = 9) [Color figure can be viewed at wileyonlinelibrary.com]

We next focused on the specific subsets of orthologous genes that possibly constitute the signaling dependencies of developmental burden. If developmental burden works only on actual signaling cascades that make up its dependencies, it is possible that conservation force could be limited to these gene sets rather than the overall gene expression profile. In this regard, the sonic hedgehog signaling cascade would be an attractive candidate to detect conservation, as this signaling molecule, secreted from the notochord and the floor plate of the neural tube, is necessary for both neural tube and somite differentiation (Gilbert, 2014). In accordance, we found that the expression of *shh* in the posterior notochord of turtle and chicken was significantly conserved (fewer expression changes between species) than those of genomic background (*p* = .001, Wilcoxon rank sum test). In the posterior notochord, we also found a weak sign of similar expression (within twofold ratio between turtle and chicken) for shh‐related genes as a whole (46 genes) than genes in the genomic background (Figure 3 and Table S2). Although the enrichment was not drastic (only 1.4‐times higher ratio than the genomic background), the weak sign of conservation was also found for the shh‐related genes in the anterior notochord (Figure S2). Notably, a similar sign of conservation was also found for genes expressed in the neural tube and posterior somites (Figure S3), including genes important for receiving shh signals, such as the *septin 2‐like* (Q. Hu et al., 2010) and *tectonic 3* (Wang et al., 2018) genes. Septin 2, for example, is a membrane protein known as a diffusion barrier at the base of primary cilia that keeps the shh receptor Smo from diffusing away from the cilia (Briscoe & Therond, 2013). These results indicate that targets of evolutionary conservation are not only confined to the shh molecule itself but also include molecules related to the shh signaling cascade expressed in the surrounding structures. Nevertheless, it has to be noted that not all the shh‐related genes showed the signs of conservation at mRNA level, with anterior somites, for example, showing no statistical significance for the shh‐related gene set as a whole (Figure S3). This could be explained by the reduced effect of developmental burden in these well‐differentiated anterior somites; however, it is also possible that differences between the developmental timetable or heterochronic shifts of somites and their derivatives in turtles and chicken led to this discrepancy.

### 3.3 Potential target of developmental burden

**Figure 4:**
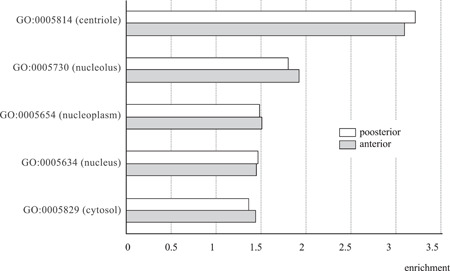
GO terms enriched in notochord–neural tube–somites conserved genes. 1:1 orthologous genes with conserved expression levels (within twofold change between turtles and chicken) in the notochord, neural tube, and somites were identified, and then subsets of genes that show conserved expressions in the late embryonic phase (TK27 for turtles and HH38 for chicken) were subtracted. These genes were further analyzed for the enrichment of GO terms by comparing them to those of genomic background, and their effect sizes are shown in the bar plot. Only GO terms with statistical significance (two‐sided Fisher's exact test with Holm‐corrected alpha levels), both in anterior and posterior structures, are shown (see also Tables S3 and S4 for more detail). The *X* axis represents times enrichment over genomic frequency

Finally, we extracted features of genes that show similar expression levels specifically within the target structures to find hints for the target of developmental burden. To avoid detecting constitutively expressed genes, such as house‐keeping genes, we excluded genes that also showed conserved levels in the late stages of chicken (whole embryo of HH38) and turtle (whole embryo of TK27). We first looked for enriched GO‐slim categories of 1:1 orthologous genes that are specifically conserved in the notochord (see Supporting Information Data S2 for the list of genes, and Supporting Information Data S3 and S4 for the GO term analysis); however, no GO‐slim terms showed consistent enrichment in both anterior and posterior notochords (Supporting Information Data S3). The tendency was essentially the same for the analysis with ortholog group expression data, as no GO‐slim term showed consistent significance for all the datasets (Supporting Information Data S4). These could be due to the pleiotropic expression of conserved genes in various tissues, including tissues in later developmental stages, as conserved genes are known to be expressed in several developmental stages, including adult organs (Song et al., 2020). We then looked for GO terms enriched in genes conserved among the notochord, neural tube, and somites, as genes that constitute the signaling dependencies of developmental burden are expected to be expressed both in the source and receiving tissues. From the genes conserved in all three tissues, we excluded genes that showed conserved expression in the latest phase of turtle (TK23) and chicken (HH38) development to avoid detecting constitutive active genes such as housekeeping genes. The results indicated that the highest enrichment (around four times) was in the GO term 0005814 (centriole), and this was true for both the anterior and posterior structures (Figure 4, Tables S3 and S4). Although the GO term 0005814 (centriole) was not found to be statistically significant with the ortholog group‐based expression data (Figure S5), overall tendencies (detected GO terms and effect sizes) were similar to the GO‐slim analysis. The reason for the enrichment of GO term centriole is not self‐evident; however, one possibility is that genes involved in functional signaling through primary cilia contributed to this enrichment. In accordance with this, we found genes, such as *Polo‐like Kinase 1* or *Plk1* (Zhang et al., 2019), *PCM1* (G. Wang, Chen, et al., 2013), *KIF2A* (Miyamoto et al., 2015), and *SIRT2* (Zhou et al., 2014), in these target subsets (Figure S4 and Supporting Information Data S5). Plk1, for example, is reportedly involved in primary cilia disassembly before mitotic entry (G. Wang, Chen, et al., 2013), as well as in the Hedgehog signaling pathway (Zhang et al., 2019).

## 4 DISCUSSION

In this study, we indirectly tested the possible existence of developmental burden by estimating the evolutionary conservation of genes expressed in structures assumed to be under the strong burden, namely, the vertebrate notochord (Figure 1). While the overall gene expression profile of notochord did not show higher conservation than in the somites and neural tube (Figure 2), the expression levels of shh and some shh‐related and centriole‐related genes were relatively conserved compared with those of genomic background (Figure 3). Notably, this sign of conservation was found not only in the notochord but also in the surrounding structures (Figures S3 and S4). These results imply that developmental burden if it exists, contributes to evolutionary conservation not only in the source of inductive signals but also in the receiving cascades in surrounding structures, which possibly constitute the inter‐dependencies of developmental burden. In other words, the term developmental burden often suggests that the most upstream structures or signals become the target of conservation. However, our results highlight that inter‐dependencies or “chains” might be the actual target of conservation. Furthermore, our results do not contradict the idea that developmental burden could be one of the mechanisms behind the strict conservation of anatomical connectivity, or phylotype, at this mid‐embryonic phase (Duboule, 1994; Richardson et al., 1998). This also coincides well with the observation that the notochord and surrounding structures are lost altogether in tailless ascidians. However, it has to be noted that our results do not necessarily support the existence of developmental burden nor any resulting direct effect. To be specific, our study did not directly measure the strength of dependencies predicted by the developmental burden, and the evolutionary conservation we detected could be due to any other effects, such as adaptation, genetic drift, and/or a pleiotropic constraint, working on repeatedly recruited genes (Galis, 1999; H. Hu et al., 2017). In addition, no consensus has been reached regarding how distantly related species should be of the target to evaluate the effect of developmental burden. Therefore, studies addressing a similar approach applied to two or more different species harboring various evolutionary distances are warranted to test our conclusion. Moreover, our research was not designed to detect actual molecular interactions between the genes that possibly constitute the inter‐dependencies. Thus, further detailed studies are warranted to evaluate the “chains” of developmental signals. Evaluation of spatio‐temporal dependencies between cells by single‐cell RNAseq technology would provide a basis for testing the concept of developmental burden and hints for understanding general relationships in embryonic evolution.

## CONFLICT OF INTERESTS

The authors declare that there are no conflict of interests.

## AUTHOR CONTRIBUTIONS

Naoki Irie and Shigehiro Kuraku conceived the study. Satoko Fujimoto and Kaori Yamanaka performed a sampling of embryos, laser microdissection experiments, and library preparations for RNAseq. Chiharu Tanegashima, Osamu Nishimura, and Shigehiro Kuraku supported RNAseq and quality check of RNAseq reads. Bioinformatics analyses were performed by Naoki Irie. Naoki Irie and Shigehiro Kuraku drafted the manuscript.

### PEER REVIEW

The peer review history for this article is available at https://publons.com/publon/10.1002/jez.b.23032

## Supporting information

Supporting information.

Supporting information.

Supporting information.

Supporting information.

Supporting information.

Supporting information.

## Data Availability

The RNAseq data are available through the DRA or SRA database with accession ID PRJDB7202 (BioProject ID).
