# Supplementary material for: Measuring potential effects of the developmental burden associated with the vertebrate notochord
Source: J Exp Zool B Mol Dev Evol. 2021 Mar 10;338(1-2):129–36. doi: 10.1002/jez.b.23032 (PMC9291948; doi:10.1002/jez.b.23032)
Supplement: Supplementary file 1 — Supporting information. [file JEZ-338-129-s003.docx]

**Measuring potential effects of developmental burden associated with the vertebrate notochord**

**Supplementary Information**

Satoko Fujimoto^1^, Kaori Yamanaka^1^, Chiharu Tanegashima^1^, Osamu Nishimura^1^, Shigehiro Kuraku^1^, Shigeru Kuratani^1^, Naoki Irie^2, 3*^

**Affiliations:**

^1^ RIKEN, Center for Biosystems Dynamics Research, Japan.

^2^ The University of Tokyo, Department of Biological Sciences

^3^ Universal Biology Institute, The University of Tokyo, Japan

*Correspondence: Naoki Irie, irie@bs.s.u-tokyo.ac.jp

Department of Biological Sciences, University of Tokyo, 7‑3‑1 Hongo,

Bunkyo‑ku, Tokyo 113‑0033, Japan

**Supplementary Table 1** RNAseq reads obtained and analyzed in this study

| **Sample_name** | **Total_reads** | **Mapped reads** | **Mapping Ratio** | **Detected (>0 TPM) genes / total genes** |
| --- | --- | --- | --- | --- |
| Gg_Somite_3p | 52255109 | 36867930 | 70.55% | 18781 / 24356 |
| Gg_Somite_3a | 55931258 | 42063522 | 75.21% | 18729 / 24356 |
| Gg_Somite_2p | 64497600 | 47891187 | 74.25% | 18664 / 24356 |
| Gg_Somite_2a | 57858792 | 43465449 | 75.12% | 18951 / 24356 |
| Gg_Somite_1p | 56242319 | 41824207 | 74.36% | 18347 / 24356 |
| Gg_Somite_1a | 59640358 | 44854746 | 75.21% | 19084 / 24356 |
| Gg_Notochord_3p | 53613251 | 39307507 | 73.32% | 17749 / 24356 |
| Gg_Notochord_3a | 55925980 | 41875238 | 74.88% | 16662 / 24356 |
| Gg_Notochord_2p | 57434183 | 41217753 | 71.77% | 16539 / 24356 |
| Gg_Notochord_2a | 59140198 | 43652271 | 73.81% | 16589 / 24356 |
| Gg_Notochord_1p | 59219970 | 42725242 | 72.15% | 16918 / 24356 |
| Gg_Notochord_1a | 62203984 | 46035453 | 74.01% | 16161 / 24356 |
| Gg_NeuralTube_3p | 51221594 | 34820141 | 67.98% | 18974 / 24356 |
| Gg_NeuralTube_3a | 57132288 | 43340978 | 75.86% | 17773 / 24356 |
| Gg_NeuralTube_2p | 57406762 | 42617740 | 74.24% | 19067 / 24356 |
| Gg_NeuralTube_2a | 55991230 | 41777287 | 74.61% | 17827 / 24356 |
| Gg_NeuralTube_1p | 56403861 | 41825105 | 74.15% | 19044 / 24356 |
| Gg_NeuralTube_1a | 55887804 | 42226211 | 75.56% | 16737 / 24356 |
| Gg_HH38_1 | 31726995 | 21200228 | 66.82% | 17006 / 24356 |
| Gg_HH38_2 | 31012093 | 20641954 | 66.56% | 16861 / 24356 |
| Ps_Somite_3p | 51162738 | 39111694 | 76.45% | 14384 / 19328 |
| Ps_Somite_3a | 52426463 | 40684027 | 77.60% | 14484 / 19328 |
| Ps_Somite_2p | 43329995 | 31004503 | 71.55% | 13557 / 19328 |
| Ps_Somite_2a | 51692830 | 38909871 | 75.27% | 14412 / 19328 |
| Ps_Somite_1p | 49452817 | 36894767 | 74.61% | 13573 / 19328 |
| Ps_Somite_1a | 43780556 | 31891588 | 72.84% | 13943 / 19328 |
| Ps_Notochord_3p | 56670076 | 43470450 | 76.71% | 13937 / 19328 |
| Ps_Notochord_3a | 52075600 | 39664217 | 76.17% | 13515 / 19328 |
| Ps_Notochord_2p | 46299137 | 31755710 | 68.59% | 12874 / 19328 |
| Ps_Notochord_2a | 56442338 | 41436324 | 73.41% | 13673 / 19328 |
| Ps_Notochord_1p | 52835178 | 39107662 | 74.02% | 13034 / 19328 |
| Ps_Notochord_1a | 57176547 | 41216561 | 72.09% | 13770 / 19328 |
| Ps_NeuralTube_3p | 49090763 | 37900536 | 77.21% | 14088 / 19328 |
| Ps_NeuralTube_3a | 51980872 | 39723480 | 76.42% | 14281 / 19328 |
| Ps_NeuralTube_2p | 45941033 | 32292205 | 70.29% | 13515 / 19328 |
| Ps_NeuralTube_2a | 59661162 | 43571183 | 73.03% | 14634 / 19328 |
| Ps_NeuralTube_1p | 49660350 | 36681175 | 73.86% | 13789 / 19328 |
| Ps_NeuralTube_1a | 47594996 | 34998175 | 73.53% | 14122 / 19328 |
| Ps_TK27_1 | 44117301 | 29134471 | 66.04% | 15587 / 19328 |
| Ps_TK27_2 | 41419511 | 24954808 | 60.25% | 15428 / 19328 |

**Supplementary Table 2** Turtle and chicken ensembl gene IDs of shh-related genes

| **1:1 ortholog pair of turtle and chicken (Ensembl IDs)** | **Gene name** |
| --- | --- |
| ENSPSIG00000017804 -- ENSGALG00000040266 | GLI3 |
| ENSPSIG00000014462 -- ENSGALG00000005707 | Intraflagellar transport 20 |
| ENSPSIG00000016273 -- ENSGALG00000015356 | Intraflagellar transport 57 |
| ENSPSIG00000018115 -- ENSGALG00000015412 | ADP ribosylation factor like GTPase 13B |
| ENSPSIG00000005251 -- ENSGALG00000011442 | Transforming growth factor beta receptor 2 |
| ENSPSIG00000016765 -- ENSGALG00000015029 | EvC ciliary complex subunit 2 |
| ENSPSIG00000005108 -- ENSGALG00000015037 | EvC ciliary complex subunit 1 |
| ENSPSIG00000009951 -- ENSGALG00000009559 | Intraflagellar transport 80 |
| ENSPSIG00000015313 -- ENSGALG00000012792 | Homeodomain interacting protein kinase 2 |
| ENSPSIG00000002314 -- ENSGALG00000015152 | BOC cell adhesion associated, oncogene regulated |
| ENSPSIG00000010417 -- ENSGALG00000010478 | SCL/TAL1 interrupting locus |
| ENSPSIG00000002938 -- ENSGALG00000038608 | Heat shock protein family B (small) member 11 |
| ENSPSIG00000016979 -- ENSGALG00000006379 | Sonic hedgehog |
| ENSPSIG00000006958 -- ENSGALG00000036114 | Smoothened, frizzled class receptor |
| ENSPSIG00000004046 -- ENSGALG00000001141 | Hes family bHLH transcription factor 5 |
| ENSPSIG00000009820 -- ENSGALG00000015208 | Receptor tyrosine kinase like orphan receptor 2 |
| ENSPSIG00000013175 -- ENSGALG00000026491 | Transmembrane protein 17 |
| ENSPSIG00000013662 -- ENSGALG00000008866 | WD repeat containing planar cell polarity effector |
| ENSPSIG00000006746 -- ENSGALG00000011880 | Bardet–Biedl syndrome 7 |
| ENSPSIG00000011075 -- ENSGALG00000006860 | Kinesin family member 3A |
| ENSPSIG00000003724 -- ENSGALG00000001939 | Homeodomain interacting protein kinase 1 |
| ENSPSIG00000009707 -- ENSGALG00000014514 | Coiled-coil and C2 domain containing 2A |
| ENSPSIG00000008386 -- ENSGALG00000012620 | Patched 1 |
| ENSPSIG00000015910 -- ENSGALG00000005128 | Protein tyrosine phosphatase domain containing 1 |
| ENSPSIG00000016252 -- ENSGALG00000003523 | Intraflagellar transport 52 |
| ENSPSIG00000010749 -- ENSGALG00000005096 | B9 domain containing 1 |
| ENSPSIG00000010761 -- ENSGALG00000035419 | Cell adhesion associated, oncogene regulated |
| ENSPSIG00000016909 -- ENSGALG00000012025 | TALPID3 |
| ENSPSIG00000013841 -- ENSGALG00000017148 | Centromere protein J |
| ENSPSIG00000003938 -- ENSGALG00000008049 | ADP ribosylation factor like GTPase 3 |
| ENSPSIG00000015250 -- ENSGALG00000036869 | NK2 homeobox 2 |
| ENSPSIG00000004186 -- ENSGALG00000014876 | TBC1 domain family member 32 |
| ENSPSIG00000013232 -- ENSGALG00000011847 | Tetratricopeptide repeat domain 26 |
| ENSPSIG00000009877 -- ENSGALG00000002504 | TROVE domain family member 2 |
| ENSPSIG00000014564 -- ENSGALG00000016358 | Patched domain containing 1 |
| ENSPSIG00000002175 -- ENSGALG00000016895 | DAZ interacting zinc finger protein 1 |
| ENSPSIG00000002946 -- ENSGALG00000001652 | Septin 2-like |
| ENSPSIG00000014096 -- ENSGALG00000030979 | Meckel syndrome, type 1 |
| ENSPSIG00000010828 -- ENSGALG00000007493 | NAD(P) dependent steroid dehydrogenase-like |
| ENSPSIG00000006256 -- ENSGALG00000009881 | Hedgehog acyltransferase |
| ENSPSIG00000016840 -- ENSGALG00000007438 | Intraflagellar transport 46 |
| ENSPSIG00000010009 -- ENSGALG00000042990 | Intraflagellar transport 27 |
| ENSPSIG00000010796 -- ENSGALG00000003219 | Transmembrane protein 231 |
| ENSPSIG00000011875 -- ENSGALG00000010956 | Tetratricopeptide repeat domain 21B |
| ENSPSIG00000005208 -- ENSGALG00000016509 | Intraflagellar transport 172 |
| ENSPSIG00000014461 -- ENSGALG00000010517 | Tectonic family member 3 |

**Supplementary Table 3** GO enrichment analysis for 1:1 orthologous genes conserved in posterior Notochord-Neural Tube-Somites after subtracting gene sets conserved in the late stage of turtle and chicken

| **GO term ID** | **GO term description** | **Count** | **Total count in the subset** | **count in genome** | **Total GO terms in genome** | **Effect size** | **p-value** |
| --- | --- | --- | --- | --- | --- | --- | --- |
| GO:0005814 | centriole | 23 | 14,186 | 64 | 126,672 | 3.21 | 1.21 × 10^−5^ |
| GO:0005813 | centrosome | 64 | 14,186 | 268 | 126,672 | 2.13 | 5.54 × 10^−7^ |
| GO:0005730 | nucleolus | 72 | 14,186 | 357 | 126,672 | 1.80 | 1.82 × 10^−5^ |
| GO:0005654 | nucleoplasm | 174 | 14,186 | 1,050 | 126,672 | 1.48 | 5.63 × 10^−6^ |
| GO:0005829 | cytosol | 249 | 14,186 | 1,523 | 126,672 | 1.46 | 1.54 × 10^−7^ |
| GO:0005634 | nucleus | 408 | 14,186 | 2,686 | 126,672 | 1.36 | 4.77 × 10^−8^ |
| GO:0005886 | plasma membrane | 105 | 14,186 | 1,398 | 126,672 | 0.67 | 4.01 × 10^−5^ |
| GO:0016020 | membrane | 204 | 14,186 | 3,398 | 126,672 | 0.54 | 7.45 × 10^−21^ |
| GO:0016021 | integral component of membrane | 153 | 14,186 | 2,958 | 126,672 | 0.46 | 3.83 × 10^−25^ |
| GO:0007165 | signal transduction | 26 | 14,186 | 669 | 126,672 | 0.35 | 5.83 × 10^−10^ |
| GO:0005887 | integral component of plasma membrane | 21 | 14,186 | 559 | 126,672 | 0.34 | 9.47 × 10^−9^ |
| GO:0055085 | transmembrane transport | 13 | 14,186 | 363 | 126,672 | 0.32 | 2.67 × 10^−6^ |
| GO:0005509 | calcium ion binding | 13 | 14,186 | 380 | 126,672 | 0.31 | 7.96 × 10^−7^ |
| GO:0005615 | extracellular space | 15 | 14,186 | 516 | 126,672 | 0.26 | 2.68 × 10^−10^ |
| GO:0006811 | ion transport | 7 | 14,186 | 272 | 126,672 | 0.23 | 1.93 × 10^−6^ |
| GO:0005576 | extracellular region | 10 | 14,186 | 395 | 126,672 | 0.23 | 4.17 × 10^−9^ |
| GO:0007186 | G protein-coupled receptor signaling pathway | 5 | 14,186 | 353 | 126,672 | 0.13 | 5.00 × 10^−11^ |
| GO:0004930 | G protein-coupled receptor activity | 2 | 14,186 | 290 | 126,672 | 0.06 | 3.38 × 10^−11^ |

**Supplementary Table 4** GO enrichment analysis for 1:1 orthologous genes conserved in anterior Notochord-Neural Tube-Somites after subtracting gene sets conserved in late turtle and chicken embryos

| **GO term ID** | **GO term description** | **Count** | **Total count in the subset** | **count in genome** | **Total GO terms in genome** | **Effect size** | **p-value** |
| --- | --- | --- | --- | --- | --- | --- | --- |
| GO:0005814 | centriole | 21 | 13,442 | 64 | 126,630 | 3.09 | 4.14 × 10^−5^ |
| GO:0005730 | nucleolus | 73 | 13,442 | 358 | 126,630 | 1.92 | 2.39 × 10^−6^ |
| GO:0005654 | nucleoplasm | 169 | 13,442 | 1,054 | 126,630 | 1.51 | 2.18 × 10^−6^ |
| GO:0005634 | nucleus | 410 | 13,442 | 2,676 | 126,630 | 1.44 | 6.03 × 10^−11^ |
| GO:0005829 | cytosol | 233 | 13,442 | 1,530 | 126,630 | 1.44 | 1.03 × 10^−6^ |
| GO:0005886 | plasma membrane | 90 | 13,442 | 1,406 | 126,630 | 0.603 | 8.54 × 10^−7^ |
| GO:0016020 | membrane | 204 | 13,442 | 3,393 | 126,630 | 0.566 | 2.48 × 10^−17^ |
| GO:0016021 | integral component of membrane | 155 | 13,442 | 2,958 | 126,630 | 0.494 | 4.59 × 10^−21^ |
| GO:0005615 | extracellular space | 21 | 13,442 | 514 | 126,630 | 0.385 | 9.77 × 10^−7^ |
| GO:0007165 | signal transduction | 25 | 13,442 | 667 | 126,630 | 0.353 | 2.65 × 10^−9^ |
| GO:0005887 | integral component of plasma membrane | 20 | 13,442 | 560 | 126,630 | 0.336 | 2.42 × 10^−8^ |
| GO:0006811 | ion transport | 8 | 13,442 | 271 | 126,630 | 0.278 | 2.31 × 10^−5^ |
| GO:0005576 | extracellular region | 11 | 13,442 | 394 | 126,630 | 0.263 | 1.04 × 10^−7^ |
| GO:0007186 | G protein-coupled receptor signaling pathway | 4 | 13,442 | 354 | 126,630 | 0.106 | 3.28 × 10^−11^ |
| GO:0004930 | G protein-coupled receptor activity | 2 | 13,442 | 290 | 126,630 | 0.064 | 1.87 × 10^−10^ |

**Supplementary Figure 1: Evolutionary conservation of notochord, neural tube, and somites between chicken and turtles by ortholog group-based method.**

Evolutionary distances of gene expression profiles were evaluated by 1 – spearman correlation coefficients of ortholog-groups between turtles and chicken. Ortholog groups between turtles and chicken were identified by the orthoMCL program, and the 1 – spearman correlation coefficient was calculated as previously described (Hu *et al*., 2017). **(a)** Expression levels of paralogous genes were summed to estimate the expression levels of each ortholog-group. **(b)** Mean expression levels of paralogous genes were used to estimate the expression levels of each ortholog group. Bar plots on the left represent sections from the anterior level, and bar plots on the right represent sections from the posterior level. N=3. Different structures were dissected from the same individual for each biological replicate. Error bar: S.D. p values: Dunnet test (two-tailed).


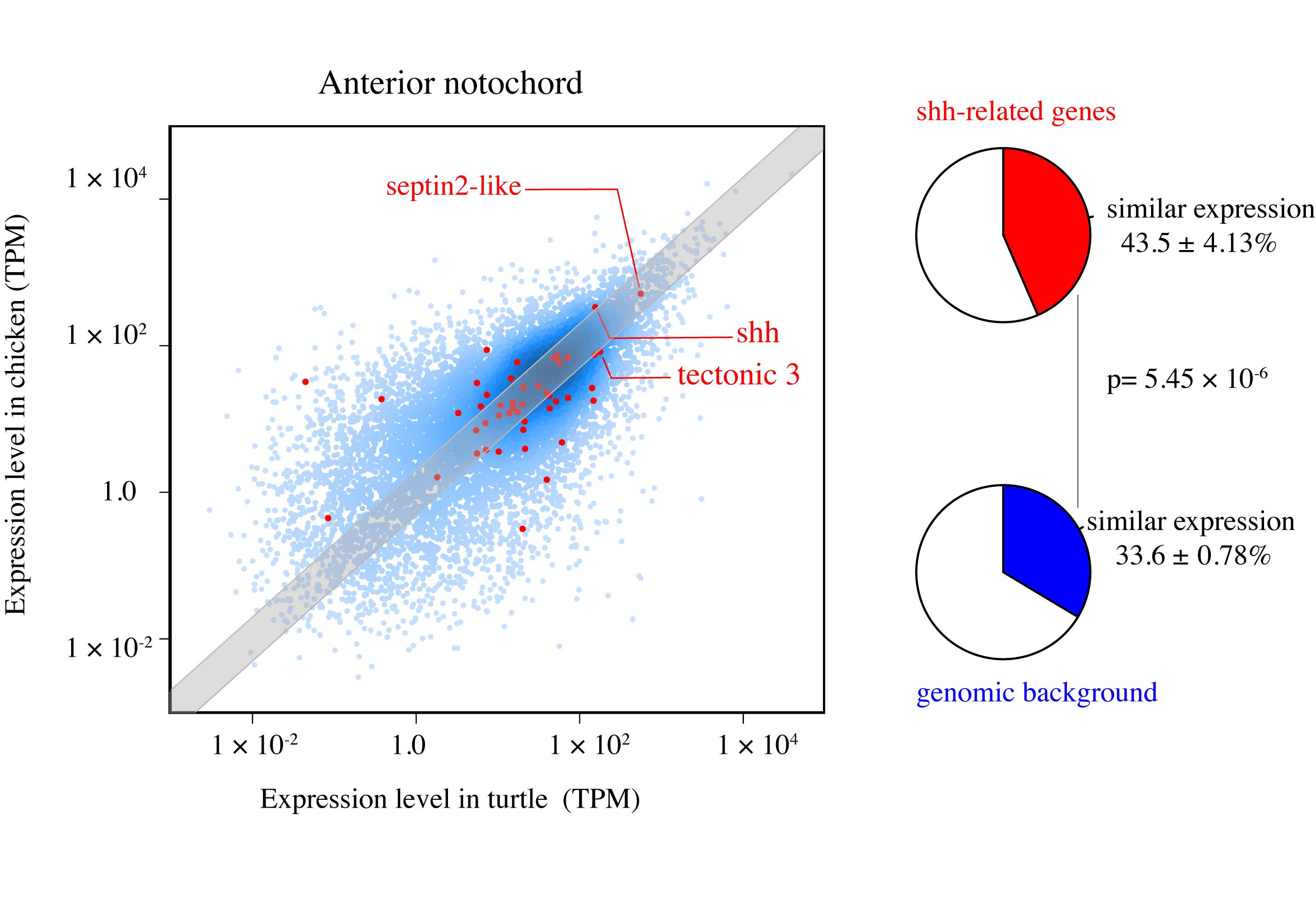


**Supplementary Figure 2: Conserved expression levels of shh-related genes in the posterior notochord**

**Left**. Gene expression levels (TPM) in posterior notochords of chicken and turtles are shown as a scatter plot (left). Shh-related genes (46 genes) are colored in red, and other genes in the genomic background are colored in light blue (12233 genes). Each plot represents average expression levels of biological replicates within each species. The gray zone represents signal ratio chicken-turtle less than 2-fold. **Right**. Pie charts represent the ratio of shh-related genes within the 2-fold range (up), and the ratio of the genomic background (down). Deviations in ratios represent S.D. The differences in ratio of genes within the 2-fold range and genes outside the 2-fold range were statistically significant between shh-related genes and genomic background (student-t test, n=9).


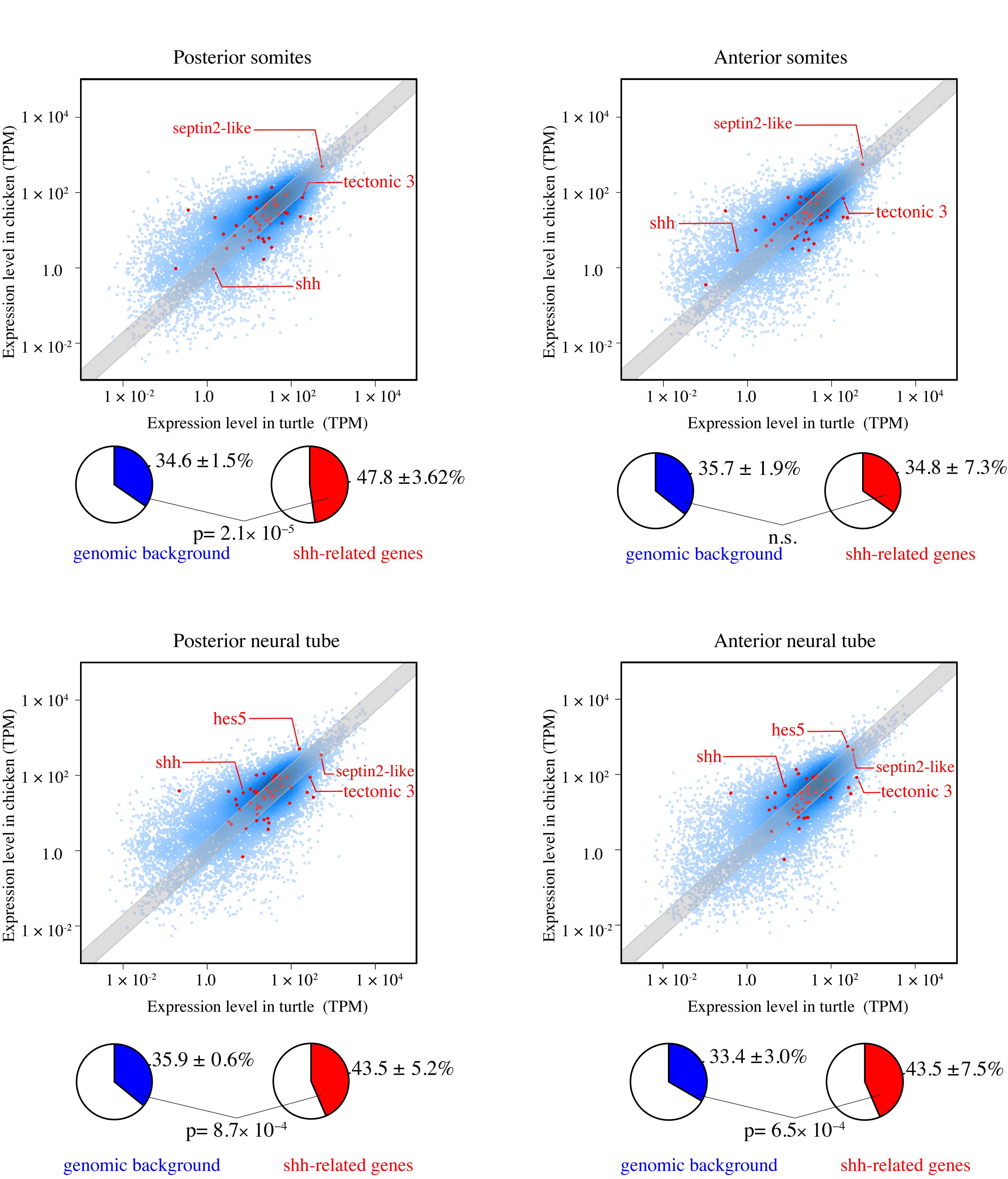


**Supplementary Figure 3 Expression levels of shh-related genes in the somites and neural tube**

Gene expression levels (TPM) in the anterior/posterior somites and neural tube of chicken and turtles are shown as a scatter plot. Shh-related genes (46 genes) are colored in red, and other genes in the genomic background are colored in light blue (12233 genes). Each plot represents average expression levels of biological replicates. The gray zone represents signal ratio chicken-turtle less than 2-fold. Pie charts represent the ratio of shh-related genes within the 2-fold range (red), and the ratio of genomic background (blue). Deviations in ratios represent S.D. The differences in ratio of genes within the 2-fold range and genes outside the 2-fold range were statistically significant between shh-related genes and the genomic background (student-t test, n=9).


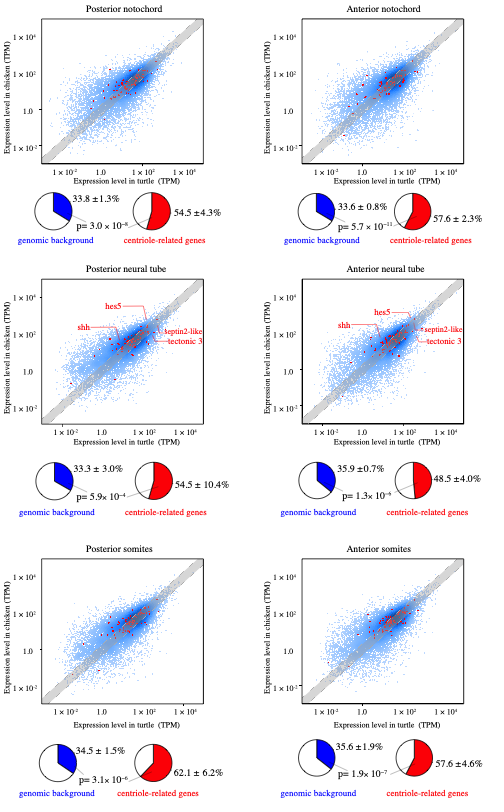


**Supplementary Figure 4: Expression levels of centriole-related genes in the somites and neural tube**

Gene expression levels (TPM) in anterior/posterior somites and neural tube of chicken and turtles are shown as a scatter plot. Centriole-related genes (defined as genes having GO:0005814, 66 genes in total) are colored in red, and other genes in the genomic background are colored in light blue (12213 genes). Each plot represents average expression levels of biological replicates. The gray zone represents signal ratio chicken-turtle less than 2-fold. Pie charts represent the ratio of centriole-related genes within the 2-fold range (red), and the ratio of genomic background (blue). Deviations in ratios represent S.D. The differences in the ratio of genes within the 2-fold range and genes outside the 2-fold range were statistically significant between shh-related genes and the genomic background (student-t test, n=9).

**Supplementary Figure 5: GO terms enriched in notochord-neural tube-somites conserved genes**

Ortholog groups (OGs) with conserved expression (within 2-fold change between turtles and chicken) in the notochord, neural tube, and somites were identified, and then subsets of OGs that showed conserved expression in late embryonic phase (TK27 for turtles and HH38 for chicken) were subtracted. These OGs were further analyzed for the enrichment of GO terms by comparison to those of genomic background, and their effect sizes are shown in the bar plot. GO terms with statistical significance (two-sided Fisher’s exact test with Holm corrected alpha levels), both in anterior and posterior structures, are shown (except for GO:0005814 Centriole, as this term was not statistically significant after alpha-level correction for the multiple-comparisons. P-values are shown for each data). The X axis represents times enrichment over genomic frequency.

**Supplementary Data 1:**

Gene expression table of 1:1 orthologs in targeted structures

**Supplementary Data 2:**

Chicken gene IDs conserved both in the anterior and posterior notochord but not in neural tube nor somites

**Supplementary Data 3:**

Result of GO-slim enrichment analysis for 1:1 ortholog genes specifically conserved in notochord

**Supplementary Data 4:**

Result of GO-slim enrichment analysis for ortholog groups specifically conserved in notochord

**Supplementary Data 5:**

Relative expression levels of centriole related genes

**REFERENCES**

Abzhanov A. (2013). von Baer's law for the ages: lost and found principles of developmental evolution. *Trends Genet*, *29*, 712-722.

Alan, C. L. (2014). *Conceptual change in biology : scientific and philosophical perspectives on evolution and development*. New York: Springer.

Briscoe, J., & Therond, P. P. (2013). The mechanisms of Hedgehog signalling and its roles in development and disease. *Nature reviews. Molecular cell biology, 14*(7), 416-429. doi:10.1038/nrm3598

Domazet-Loso, T., & Tautz, D. (2010). A phylogenetically based transcriptome age index mirrors ontogenetic divergence patterns. *Nature, 468*(7325), 815-818. doi:10.1038/nature09632

Duboule, D. (1994). Temporal colinearity and the phylotypic progression: a basis for the stability of a vertebrate Bauplan and the evolution of morphologies through heterochrony. *Development*, 135-142.

Galis, F. (1999). Why do almost all mammals have seven cervical vertebrae? Developmental constraints, Hox genes, and cancer. *The Journal of experimental zoology, 285*(1), 19-26.

Garstang, W. (1922). The theory of recapitulation: a critical re-statement of the biogenetic law. *Zool J Linnean Soc., 35*, 81–101.

Gilbert., S. F. (2014). *Developmental Biology*: Sinauer Associates, Inc.

Haeckel, E. (1866). *Generelle Morphologie der Organismen: Allgemeine Grundzuge der organischen Formen-Wissenschaft, mechanisch begrundet durch die von Charles Darwin reformirte Descendenz-Theorie.* : Georg Reimer.

Hazkani-Covo, E., Wool, D., & Graur, D. (2005). In search of the vertebrate phylotypic stage: a molecular examination of the developmental hourglass model and von Baer's third law. *Journal of experimental zoology. Part B, Molecular and developmental evolution, 304*(2), 150-158. doi:10.1002/jez.b.21033

Hu, H., Uesaka, M., Guo, S., Shimai, K., Lu, T. M., Li, F., . . . Consortium, E. (2017). Constrained vertebrate evolution by pleiotropic genes. *Nat Ecol Evol, 1*(11), 1722-1730. doi:10.1038/s41559-017-0318-0

Hu, Q., Milenkovic, L., Jin, H., Scott, M. P., Nachury, M. V., Spiliotis, E. T., & Nelson, W. J. (2010). A septin diffusion barrier at the base of the primary cilium maintains ciliary membrane protein distribution. *Science, 329*(5990), 436-439. doi:10.1126/science.1191054

Irie, N. (2017). Remaining questions related to the hourglass model in vertebrate evolution. *Curr Opin Genet Dev, 45*, 103-107. doi:10.1016/j.gde.2017.04.004

Irie, N., & Kuratani, S. (2011). Comparative transcriptome analysis reveals vertebrate phylotypic period during organogenesis. *Nature communications, 2*, 248. doi:10.1038/ncomms1248

Irie, N., & Kuratani, S. (2014). The developmental hourglass model: a predictor of the basic body plan? *Development, 141*(24), 4649-4655. doi:10.1242/dev.107318

Irie, N., Satoh, N., & Kuratani, S. (2018). The phylum Vertebrata: a case for zoological recognition. *Zoological Lett, 4*, 32. doi:10.1186/s40851-018-0114-y

Irie, N., & Sehara-Fujisawa, A. (2007). The vertebrate phylotypic stage and an early bilaterian-related stage in mouse embryogenesis defined by genomic information. *BMC biology, 5*, 1. doi:10.1186/1741-7007-5-1

Jeffery, W. R., & Swalla, B. J. (1992). Evolution of alternate modes of development in ascidians. *BioEssays, 14*(4), 219-226. doi:10.1002/bies.950140404

Kalinka, A. T., & Tomancak, P. (2012). The evolution of early animal embryos: conservation or divergence? *Trends Ecol Evol, 27*(7), 385-393. doi:10.1016/j.tree.2012.03.007

Kalinka, A. T., Varga, K. M., Gerrard, D. T., Preibisch, S., Corcoran, D. L., Jarrells, J., . . . Tomancak, P. (2010). Gene expression divergence recapitulates the developmental hourglass model. *Nature, 468*(7325), 811-814. doi:10.1038/nature09634

Kim, D., Paggi, J. M., Park, C., Bennett, C., & Salzberg, S. L. (2019). Graph-based genome alignment and genotyping with HISAT2 and HISAT-genotype. *Nat Biotechnol, 37*(8), 907-915. doi:10.1038/s41587-019-0201-4

Kovaka, S., Zimin, A. V., Pertea, G. M., Razaghi, R., Salzberg, S. L., & Pertea, M. (2019). Transcriptome assembly from long-read RNA-seq alignments with StringTie2. *Genome Biol, 20*(1), 278. doi:10.1186/s13059-019-1910-1

Kuratani, S. (2017). *Evolutionary Morphology: Bauplan and Embryonic Development of Vertebrates (New version)*: University of Tokyo Press.

Kuratani, S., & Ota, K. G. (2008). Primitive versus derived traits in the developmental program of the vertebrate head: views from cyclostome developmental studies. *Journal of experimental zoology. Part B, Molecular and developmental evolution, 310*(4), 294-314. doi:10.1002/jez.b.21190

Li, L., Stoeckert, C. J., Jr., & Roos, D. S. (2003). OrthoMCL: identification of ortholog groups for eukaryotic genomes. *Genome Res, 13*(9), 2178-2189. doi:10.1101/gr.1224503

Miyamoto, T., Hosoba, K., Ochiai, H., Royba, E., Izumi, H., Sakuma, T., . . . Matsuura, S. (2015). The Microtubule-Depolymerizing Activity of a Mitotic Kinesin Protein KIF2A Drives Primary Cilia Disassembly Coupled with Cell Proliferation. *Cell Rep, 10*(5), 664-673. doi:10.1016/j.celrep.2015.01.003

Nagashima H, Sugahara F, Takechi M, Ericsson R, Kawashima-Ohya Y, Narita Y, Kuratani S. (2009). Evolution of the Turtle Body Plan by the Folding and Creation of New Muscle Connections. *Science*, *325*(5937), 193-196. doi:10.1126/science.1173826

Richardson, M. K., & Keuck, G. (2002). Haeckel's ABC of evolution and development. *Biol Rev Camb Philos Soc, 77*(4), 495-528. doi:10.1017/s1464793102005948

Richardson, M. K., Minelli, A., Coates, M., & Hanken, J. (1998). Phylotypic stage theory. *Trends Ecol Evol, 13*(4), 158. doi:10.1016/s0169-5347(98)01340-8

Riedl, R. (1978). *Order in Living Organisms: A Systems Analysis of Evolution*: Wiley.

Sasagawa, Y., Nikaido, I., Hayashi, T., Danno, H., Uno, K. D., Imai, T., & Ueda, H. R. (2013). Quartz-Seq: a highly reproducible and sensitive single-cell RNA sequencing method, reveals non-genetic gene-expression heterogeneity. *Genome Biol, 14*(4), R31. doi:10.1186/gb-2013-14-4-r31

Song, J., Pineault, K., Dones J,. Raines, R., & Wellik, D. (2020). Hox genes maintain critical roles in the adult skeleton. *Proc Natl Acad Sci U S A*., *117*(13), 7296-7304. doi:10.1073/pnas.1920860117

Tanegashima, C., Nishimura, O., Motone, F., Tatsumi, K., Kadota, M., & Kuraku, S. (2018). Embryonic transcriptome sequencing of the ocellate spot skate Okamejei kenojei. *Sci Data, 5*, 180200. doi:10.1038/sdata.2018.200

Uesaka, M., Kuratani, S., Takeda, H., & Irie, N. (2019). Recapitulation-like developmental transitions of chromatin accessibility in vertebrates. *Zoological Lett, 5*, 33. doi:10.1186/s40851-019-0148-9

von Baer, K. E. (1828). *Uber Entwickelungsgeschichte der Thiere: Beobachtung und Reflektion*: Koenigsberg. Wang, B., Zhang, Y., Dong, H., Gong, S., Wei, B., Luo, M., . . . Sun, M. (2018). Loss of Tctn3 causes neuronal apoptosis and neural tube defects in mice. *Cell Death Dis, 9*(5), 520. doi:10.1038/s41419-018-0563-4

Wang, G., Chen, Q., Zhang, X., Zhang, B., Zhuo, X., Liu, J., . . . Zhang, C. (2013). PCM1 recruits Plk1 to the pericentriolar matrix to promote primary cilia disassembly before mitotic entry. *J Cell Sci, 126*(Pt 6), 1355-1365. doi:10.1242/jcs.114918

Wang, Z., Pascual-Anaya, J., Zadissa, A., Li, W., Niimura, Y., Huang, Z., . . . Irie, N. (2013). The draft genomes of soft-shell turtle and green sea turtle yield insights into the development and evolution of the turtle-specific body plan. *Nature Genet, 45*(6), 701-706. doi:10.1038/ng.2615

Wimsatt, W. C. (1986). *Integrating Scientific Disciplines (ed Bechtel, P. W.)* Springer.

Xu, F., Domazet-Loso, T., Fan, D., Dunwell, T. L., Li, L., Fang, X., & Zhang, G. (2016). High expression of new genes in trochophore enlightening the ontogeny and evolution of trochozoans. *Sci Rep, 6*, 34664. doi:10.1038/srep34664

Zhang, T., Xin, G., Jia, M., Zhuang, T., Zhu, S., Zhang, B., . . . Zhang, C. (2019). The Plk1 kinase negatively regulates the Hedgehog signaling pathway by phosphorylating Gli1. *J Cell Sci, 132*(2), jcs220384. doi:10.1242/jcs.220384

Zhou, X., Fan, L. X., Li, K., Ramchandran, R., Calvet, J. P., & Li, X. (2014). SIRT2 regulates ciliogenesis and contributes to abnormal centrosome amplification caused by loss of polycystin-1. *Hum Mol Genet, 23*(6), 1644-1655. doi:10.1093/hmg/ddt556
